# Public perspectives on health improvement within a remote‐rural island community

**DOI:** 10.1111/hex.13260

**Published:** 2021-05-06

**Authors:** Bobby Macaulay, Neil McHugh, Artur Steiner

**Affiliations:** ^1^ Yunus Centre for Social Business and Health Glasgow Caledonian University Glasgow UK

**Keywords:** health, island, public perspectives, Q methodology, rural

## Abstract

**Background:**

Rural health outcomes are often worse than their urban counterparts. While rural health theory recognizes the importance of the social determinants of health, there is a lack of insight into public perspectives for improving rural health beyond the provision of health‐care services. Gaining insight into perceived solutions, that include and go beyond health‐ care, can help to inform resource allocation decisions to improve rural health.

**Objective:**

To identify and describe shared perspectives within a remote‐rural community on how to improve rural health.

**Method:**

Using Q methodology, a set of 40 statements were developed representing different perceptions of how to improve rural health. Residents of one remote‐rural island community ranked this statement set according to their level of agreement. Card‐sorts were analysed using factor analysis to identify shared points of view and interpreted alongside post‐sort qualitative interviews.

**Results:**

Sixty‐two respondents participated in the study. Four shared perspectives were identified, labelled: Local economic activity; Protect and care for the community; Redistribution of resources; and Investing in people. Factors converged on the need to relieve poverty and ensure access to amenities and services.

**Discussion and conclusions:**

Factors represent different elements of a multifaceted theory of rural health, indicating that ‘lay’ respondents are capable of comprehending various approaches to health improvement and perspectives are not homogenous within rural communities. Respondents diverged on the role of individuals, the public sector and ‘empowered’ community‐based organizations in delivering these solutions, with implications for policy and practice.

**Public Contribution:**

Members of the public were involved in the development and piloting of the statement set.

## 1 INTRODUCTION

Rural health outcomes are often worse than their urban counterparts, especially concerning mental health, with life expectancy in ‘developed’ countries being lower in rural communities.^1^ While rurality per se is not considered to negatively affect health outcomes, the intrinsic geographical isolation of rural communities exacerbates other overarching conditions.^2^, ^3^ In response, a multifaceted approach to addressing societal conditions of ‘poverty, discrimination, inequality, [and] inequalities of resource allocation’^1^ is proposed to improve health outcomes in rural communities. A theoretical basis for understanding and analysing rural health emphasizes the influence of local cultures, amenities and health‐care services as well as their interactions with broader health systems.^2^, ^4^

The ability of local people to influence and design context‐specific health interventions, that consider local needs, can benefit rural health outcomes.^5^, ^6^ Consequently, policymakers and researchers are increasingly engaging with rural communities to coproduce solutions.^7^ Over a hundred methods of public engagement, ‘including focus groups, participatory appraisal, Planning for Real, citizen's juries and future visioning’^8^ seek to understand perspectives.^9^ Despite the proliferation of *methods* to integrate public perspectives into rural health planning, published literature exploring lay perceptions of potential rural health *solutions* is underdeveloped.^10^ The small field of literature investigating public perceptions towards rural health improvement focuses exclusively on the coproduction of rural health‐care services.^6^ For example, the Rural Service Futures (RSF) project engages rural residents in the coproduction of health services to aid evidence‐informed health decision making in remote‐rural communities.^8^, ^11^, ^12^, ^13^ RSF sought to uncover, discuss and coproduce solutions to rural and remote health‐care provision, but did not consider other means through which to improve health outcomes and reduce health inequalities.

Thus, a knowledge gap exists regarding public perspectives of the relative role of non‐health‐care means to improve rural health. This is important, as it is well established that health is determined by social, economic and environmental factors—the social determinants of health.^14^, ^15^, ^16^ Targeting these determinants can require acting further ‘upstream’ on underlying causes of poor health, rather than modifying individuals’ health behaviours through more ‘downstream’ interventions. Gaining insight into perceived solutions, that include and go beyond health‐care, can help to inform resource allocation decisions, in terms of the development and implementation of interventions and policies, to improve rural health.

The aim of this paper is to identify and describe the shared perspectives of residents of one remote‐rural island community in Scotland, on how to improve rural health. We do so by using Q methodology.

## 2 METHODS

**FIGURE 1:**
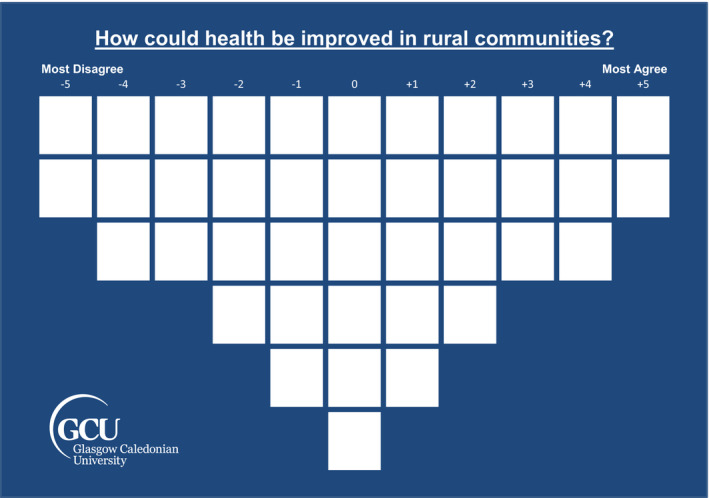
40 Statement Q grid

Q methodology is a mixed‐method comprising the collection, analysis and presentation of both quantitative and qualitative data collected by means of a card‐sorting exercise followed by a short qualitative interview. The card‐sort involves respondents’ rank‐ordering statements, typically statements of opinion, onto a quasi‐normal shaped grid according to a condition of instruction, such as from ‘Most agree’ to ‘Most disagree’ (See Figure 1). By‐person factor analysis is then used to identify patterns of similarity between the card‐sorts, known as factors. Factors represent a shared perspective on the topic in question and are represented by a distinctive ranking of the original statement set. These idealized card‐sorts are then described and interpreted, with post‐sort interview data also drawn on, to produce a rich narrative of each factor.

Q methodology is particularly well‐suited for working with ‘lay’ respondents on policy‐relevant issues and is increasingly being used in studies of public engagement in health.^10^, ^17^, ^18^, ^19^ ‘Lay’ respondents do not have to respond on the spot to open‐ended questions on how to improve health, which they may not have previously considered, as the range of options for impacting on health is presented for their consideration.^10^ This can also help to overcome issues with existing methods of public engagement in health that do not ‘adequately account for the complex value laden and holistic nature of [health service] planning, as well as for the remote and rural […] context’.^13^ Areas of agreement can also be identified among plural views. This can provide a starting point for the development of interventions and policy, particularly useful for rural communities where public engagement can be ‘messy’^6^ and disagreement among communities is common.^8^, ^20^

### 2.1 The statement set

In defining the specific cards to be sorted, statements are first drawn from the ‘concourse’, described as ‘the flow of communicability surrounding any topic’^21^ from the ‘universe of ‘statements’ so conceived for any situation or context’.^22^ In practical terms, this means gathering a wide breadth of opinion on a subject, drawing on multiple sources and types of resource if necessary.

The concourse for this study was accessed through an assessment of the ways it is claimed health can be improved in rural communities. Sources analysed included the following: peer‐reviewed articles relating to rural health theory or interventions^1^, ^2^, ^4^, ^23^, ^24^, ^25^; websites (e.g. The National Rural Health Alliance; Prevention Institute; National Organisations of State Offices for Rural Health; University of Wisconsin Population Health Institute) and grey literature (e.g. National Advisory Committee on Rural Health and Human Services, 2017; What works? Strategies to improve rural health) relating to policy and practice; data gathered through qualitative interviews with rural residents and rural health experts, conducted as part of a wider study^26^; and statements and interview data from a similar study conducted on health in low‐income urban communities.^10^ An initial 185 statements were extracted. Statements were then categorized using theories of rural health^1^, ^2^, ^4^ to ensure that extant literature was adequately represented. This list was then distilled through deleting duplicates and merging similar statements to ensure qualitative coverage and balance. The resulting draft 36 statement set was subjected to two rounds of piloting with 19 respondents in total. Pilot respondents included academics with expertise in rural health (n = 3) and Q methodology (n = 3), in addition to ‘lay’ individuals with lived experience of rural communities (n = 13). The result of these pilots was the rewording of some statements and the addition of four previously unrepresented statements (#17, #25, #39 and #40 in Table 1). The final 40 statement set is included in Table 1.

**TABLE 1:** Statement set and factor scores

	F1	F2	F3	F4
1. Increase the amount of control people have over their own lives	−1	−2	−4	0
2. Allow people to participate in local decision making	0 **C**	−1 **C**	0 **C**	1 **C**
3. Increase the tax on things that are bad for people like alcohol, sugary food and drinks or fatty food	−4 **D**	4 **D**	−1	−1
4. Cut welfare benefits	−5 **C**	−5 **C**	−5 **C**	−5 **C**
5. Raise the taxes that people pay in a fair way	−3	−4	5 **D**	−3
6. Spend more on the NHS	0	4	4	1
7. Improve broadband provision and mobile phone coverage within the community	2	0	2	−4 **D**
8. Improve transport links	4 **D**	0	0	−3 **D**
9. Enhance road safety initiatives	−2 **C**	−2 **C**	−1 **C**	−2 **C**
10. Provide spaces and opportunities for leisure, recreation and other community activities	1	0	0	3
11. Provide anonymous care and support services within the community	−1	0	−1	4 **D**
12. Have more health campaigns	−3 **D**	0	0	0
13. Improve access to health‐care services	1	3	2	4
14. Improve childcare and nursery provision	0	2	2	0
15. Improve elderly care within the community	2	5 **D**	1	2
16. Reduce social isolation and loneliness by building relationships with people	1	1	0	4 **D**
17. Reduce the price of fuel	4	2	3	−2 **D**
18. Develop local economy by providing support and incentives for businesses	3	−2	1	−2
19. Improve availability of affordable, healthy foods	1	1	5	5
20. Increase penalties for drug‐related crime	−2	4 **D**	−4	−4
21. Develop initiatives to reduce smoking	−1	0	0	1
22. Promote responsible alcohol consumption	−2 **D**	3	1	3
23. Improve access to financial products and services, such as loans and advice	−1 **D**	−4	−3	−3
24. Attract and retain young people in the community	5 **D**	2	1	2
25. Protect and preserve the natural environment	0	−3 **D**	1	1
26. Increase access to good quality education and training	5 **D**	1	3	3
27. Pursue community ownership of land, buildings or natural resources	−2	−5	−3	−2
28. Promote local culture and identity	1 **D**	−2	−2	0
29. Implement stricter health and safety standards	−4	−1	−2	−1
30. Restrict potentially harmful land uses	0	−3	−2	0
31. Increase availability of high‐quality, safe, and affordable housing	2	3	3	−1 **D**
32. Support and develop 'traditional industries', including crofting and fishing	3 **D**	−1	−3	0
33. Increase access to good jobs	4	1	2	2
34. Make sure people have enough money to pay for their basic needs like rent, food, clothing, heat for their home	3 **D**	5	4	5
35. Protect and increase local amenities, such as post offices, libraries and banks	2	1	4	−1 **D**
36. Restrict who can move into rural communities	−4	−1 **D**	−5	−5
37. Provide a forum for discussing local issues	−1	−3 **D**	−1	1
38. Develop provision of social care services for adults and young people	0	2	−2 **D**	2
39. Provide coaching sessions for good parenting	−3 **D**	−1	−1	−1
40. Pay people money to adopt healthier lifestyles	−5	−4	−4	−4

Abbreviations: C, a consensus statement; D, a distinguishing statement.

### 2.2 Setting and participants

Respondents were drawn from residents of Eriskay, South Uist and Benbecula—neighbouring small islands (connected by causeways) in the Western Isles of Scotland. These islands were selected as part of broader research into the role of community landownership in improving rural health.^26^ The South Uist Estate, which covers most of the three islands, has been owned by the resident community since 2007.^27^, ^28^

The islands are all considered ‘very remote rural’ in the Scottish Government's 8‐fold urban rural classification.^29^ The health record of the community, and that of the Western Isles more generally, is concerning. Similar to other rural areas, the islands are experiencing a widening gap between female and male life expectancies, with the latter being the second lowest in the country.^30^ Rates of coronary heart disease and hypertension are higher than anywhere else in the UK^30^ and both planned and unplanned hospital admissions are higher than the Scottish average.^31^ The Western Isles also performs poorly on a number of different indicators of deprivation when compared to other rural areas in Scotland, including having the second lowest mean equivalized household income for all Local Authority areas in Scotland,^30^ with 1 in 4 households in relative poverty.

Typically, Q studies utilize purposive sampling techniques to identify specific perspectives considered pertinent to the research.^32^ However, as the aim was to identify perspectives from within one community, a broad cross section of the community was desired. Key characteristics, for example, residency, cultural connection, crofter [a form of smallholding farming tenant with cultural significance within the region] status, occupation and language, were used to identify a diverse range of participants (see Table 2). Respondents were recruited through face‐to‐face visits to businesses and places of work, as well as private residences and public places. In Q studies, there is no set sample size. Once all relevant demographic characteristics were represented, recruitment closed when a stable set of factors were identified and the card‐sorts of new participants only confirmed existing factors.

**TABLE 2:** Sample demographic information

	Factor 1	Factor 2	Factor 3	Factor 4	Total Sample
Number of defining sorts	16	8	4	8	62
Age					
18‐30	3	0	2	4	13
31‐50	5	2	0	2	19
51‐65	6	2	1	2	22
66+	2	4	1	0	8
Gender					
Male	9	1	3	2	24
Female	7	7	1	6	38
Education					
Secondary Education	1	3	1	2	12
Further Education	10	4	1	5	31
Higher Education	5	1	2	1	19
Residency					
Benbecula	2	2	1	3	11
Iochdar and Lochcarnan (South Uist)	3	0	0	1	9
Middle District (South Uist)	3	1	1	1	12
Daliburgh, Lochboisdale and Southend (South Uist)	6	4	1	3	25
Eriskay	2	1	1	0	5
Main occupation status					
Small business owner/ management	2	1	2	0	9
Employed	11	2	2	6	40
Unemployed	1	0	0	1	3
Retired	2	5	0	0	8
Student	0	0	0	1	2
Crofter					
Yes	5	2	2	3	22
No	11	6	2	5	40
Time lived in community					
Whole life	7	4	0	2	25
Incomer (less than 10 y)	2	2	3	2	10
Incomer (more than 10 y)	4	0	0	3	13
Returner (left and came back)	3	2	1	1	14
Gaelic speaker					
Speaks Gaelic	11	5	1	4	36
Some Gaelic	3	1	1	2	10
No Gaelic	2	2	2	2	16

### 2.3 Data collection

Respondents were first read a standardized introduction to set the context of the study. They were then asked to place each statement into one of three piles depending on whether they ‘Agreed’, ‘Disagreed’ or were ‘Neutral’ about the statement, in regard to whether they thought it could improve rural health (the condition of instruction). From the ‘Agree’ pile, respondents were then instructed to select the two cards they most agreed with and place them in the +5 column (Figure 1), placing the three they next most agreed with in the +4 column and so on, until the cards were finished or the +2 column had been filled. This process was repeated from the −5 column at the other extreme of the grid reflecting the cards most disagreed with. Finally, all remaining cards, including those considered ‘neutral’ by respondents, were sorted column by column towards the middle of the grid. Following the card‐sort, a post‐sort interview was conducted. Respondents were asked to comment on how health could be improved in rural communities overall, before being asked to justify their interpretation and placement of ‘salient’ statements (ie those placed in the +5 and −5 columns). Post‐sort interviews were audio‐recorded and transcribed. Where data were collected in group settings, card‐sorts were performed individually (on separate grids) followed by a group discussion. These started with each individual first taking turns to provide reasons for their own card‐sorts before engaging in broader discussion.

### 2.4 Analysis

Data analysis used a Q methodology software package—Ken‐Q.^33^ Centroid factor extraction was followed by Varimax rotation to identify a small number of shared perspectives (factors). Preliminary analysis was based on the following criteria: (a) eigenvalue >1 and (b) at least two ‘defining’ card‐sorts i.e. a card‐sort was statistically significantly associated with a factor (*P* < .01) and was more associated with one factor than all other factors combined (e.g. it accounted for the majority of common variance). Following the interpretation of factors from different solutions, the factor solution that was the most interpretable and coherent was selected.

The interpretation of factors used quantitative and qualitative data, focusing upon, but not limited to, the assessment of: ‘salient statements’; ‘consensus statements’ (non‐significant between any pair of factors at *P* > .01); and ‘distinguishing statements’ (sorted statistically differently in one factor compared to all others at *P* < .01).^32^

This study was granted ethical approval by the University ethics board (Ref: GSBS EC 015).

## 3 RESULTS

Sixty‐two individuals participated in the study, with data collected during one field visit (Table 2). Card‐sorts were administered one‐on‐one (27 respondents) or individually in groups of up to five respondents (13 groups involving 35 respondents).

A four‐factor solution was statistically supported and yielded interpretable accounts consistent with qualitative data. Table 1 shows the idealized card‐sorts for the four factors. Thirty‐six respondents were considered ‘defining’ sorts, indicated in Table 3 by bold type and an ‘X’. Sixteen respondents were ‘mixed‐loaders’, being significantly associated with more than one factor. The remaining ten respondents were ‘null loaders’, either not being significantly associated with any factor, or not accounting for the majority of common variance.

**TABLE 3:** Factor loadings and defining sorts (X)

Respondent Number	Factor 1	Factor 2	Factor 3	Factor 4
1	0.1912	0.286	**0.4256**	**0.4192**
2	**0.7423 X**	−0.0161	0.3029	0.0029
3	0.3602	0.1357	0.1936	0.0708
4	**0.5717 X**	0.2286	−0.1516	0.1682
5	**0.4843**	0.2228	**0.4955**	**0.4084**
6	0.2318	0.2936	−0.1528	0.3174
7	0.2662	**0.5256 X**	0.0551	0.0869
8	0.0583	0.2503	−0.1489	0.0553
9	0.0596	**0.5251 X**	0.3283	−0.0212
10	**0.633 X**	0.2432	0.3247	0.2132
11	**0.6585 X**	0.2099	0.0913	0.1688
12	**0.4963 X**	0.1597	−0.0134	0.4023
13	**0.6636 X**	0.2479	0.3557	0.0441
14	−0.1023	0.3689	0.3835	**0.599 X**
15	**0.5191**	−0.0403	0.2072	**0.6488**
16	−0.1338	**0.4798**	0.2573	**0.4977**
17	0.231	**0.5263**	0.2539	**0.5279**
18	**0.4386**	0.2921	0.3704	0.0617
19	0.2795	0.2016	0.2186	0.0028
20	0.3499	0.2856	**0.5333 X**	0.1613
21	**0.7885 X**	0.0505	0.2308	0.1068
22	**0.6059 X**	0.27	−0.0874	0.0148
23	**0.4616**	0.0248	**0.5261**	0.2018
24	0.1037	0.1061	0.0392	**0.7477 X**
25	−0.046	**0.5773 X**	0.1056	0.0023
26	−0.0067	**0.521 X**	0.3019	0.3649
27	0.3545	0.223	0.1056	0.2361
28	0.1125	0.381	**0.5254 X**	−0.0705
29	**0.7277 X**	−0.0549	0.3109	0.2258
30	**0.538**	0.0276	0.0966	**0.4546**
31	0.131	0.062	**0.5095 X**	0.066
32	0.3923	0.1199	0.327	**0.5548 X**
33	**0.408**	0.2051	**0.5149**	**0.4113**
34	**0.5075**	0.0562	**0.4463**	0.2197
35	**0.4107 X**	−0.0948	0.1712	0.0467
36	**0.5896**	0.1978	**0.4142**	**0.4419**
37	0.2785	0.1002	0.3328	**0.53 X**
38	0.22	0.3476	−0.0488	**0.5984 X**
39	0.1744	**0.5559 X**	−0.0408	0.2358
40	**0.6407**	0.1992	0.2494	**0.4504**
41	0.3501	0.0815	**0.603 X**	0.2934
42	0.3794	0.0868	0.0097	**0.4733 X**
43	**0.7373 X**	−0.0556	−0.0528	0.3256
44	0.1308	**0.5023 X**	0.3946	0.2046
45	**0.589 X**	0.3221	0.3973	0.2802
46	0.1402	0.3843	0.3701	0.0168
47	0.2865	0.0145	0.074	0.3973
48	0.2217	**0.6388**	0.095	**0.41**
49	**0.4182**	0.1842	**0.5469**	0.3791
50	0.3587	0.1627	0.13	**0.4246 X**
51	−0.0025	−0.0425	0.0009	0.3855
52	−0.3045	0.3196	0.2297	**0.5015 X**
53	**0.6642**	0.2045	0.0874	**0.4549**
54	**0.4679**	0.3214	0.2502	0.391
55	**0.4307**	**0.6269**	0.1329	0.0855
56	**0.5924 X**	0.222	0.2232	−0.0997
57	**0.5152**	**0.5525**	0.2664	0.1985
58	**0.4988 X**	0.219	0.1744	0.097
59	**0.7266 X**	0.3747	0.1044	0.0052
60	0.3734	**0.5356 X**	0.2337	0.1387
61	0.1213	**0.5132 X**	0.3107	0.0256
62	**0.5997 X**	0.1267	0.1842	0.2455
% Explained variance	19	10	9	11

Note

The formula for assessing the significance threshold for factor loadings at *P* < .01 requires multiplying the standard error (SE) by 2.58^32^: $2.58\times \left(1÷\sqrt{\text{no}.\;\text{items}\;\text{in}\;Q\;\text{set}}\right)=2.58\times \left(1÷\sqrt{40}\right)=0.4079$. Card sorts with a factor loading of over 0.4079 are considered ‘significant’, indicated by bold type. Defining sorts must be significantly associated with only one factor and account for the majority of common variance (more associated with one factor than all other factors combined), indicated with an ‘X’.

The following subsections describe each factor in turn, with reference to the placement of statements and extracts from post‐sort interviews. The former is represented by the statement number followed by the column in the factor array in which it was placed (eg ‘#33, +4’ indicates that statement #33 (Table 1) was placed in the +4 column in that particular factor array). The demographic details of those defining each factor are included in Table 2, while a brief overview of each factor is outlined in Table 4.

**TABLE 4:** Brief description of factors

Factor	Overview of perceived solution to improving rural health
1. Local economic activity	Support local businesses and provide services and amenities to stimulate local economic activity and retain sustainable population.
2. Protect and care for the community	Provide health‐care services for the elderly and vulnerable, while imposing punitive measures to curb negative health behaviours and social influences.
3. Redistribution of resources	Address societal inequalities to provide for the most vulnerable by redirecting political and fiscal priorities.
4. Investing in people	Improve health behaviours by reducing social isolation and low self‐worth via a range of means including employment, care services and strengthening social bonds.

### 3.1 Factor 1‐ Local economic activity

Factor 1 respondents perceived the solution to improving health in rural communities to be in stimulating the local economy, specifically through developing local businesses (#33, +4; #18, +3; #32, +3; #35, +2). This was seen to lead to increased employment opportunities, retention of young people and the provision of adequate income, all of which were related to health outcomes within the community (#24, +5; #26, +5; #31, +2; #36, −4). Having a job was considered to lead to a number of positive health benefits, including increased physical activity, improved mental health and the ability and inclination to pursue positive health behaviours. Such behaviours, including sporting and social activities and buying healthy food, were considered prohibitively expensive within the community without paid employment (#34, +3; #10, +1; #16, +1; #19, +1; #4, −5).

If everybody’s got a decent job, they’ve got more money, they can go away from here more [on holiday], they can afford to buy the fuel, they can afford to eat well. There is this thing where if they’re not working or on the ‘dole’ [unemployment benefit] they’re more likely to be drinking, smoking, but it does make a difference having a decent job. (Respondent 2)

Respondents holding this view disagreed with measures which would stifle economic development, including taxation and regulation by local or national government. Public money was called for to be invested in overcoming infrastructural challenges within the community (#8, +4; #17, +4; #7, +2), but other public sector interventions which increased costs or regulation for local businesses were considered cumbersome, unnecessary and detrimental to local economic development (#29, −4; #5, −3; #30, 0).

We certainly don’t need any more regulation, we don’t need any more regulation about health and safety, there’s so much already that people are drowning in it, and when it gets too much you tend to ignore it or cut corners. So I’m certainly not for having any more regulation on that type of thing. (Respondent 13)

A resistance to public sector intervention was also present at an individual level, with resentment at the government attempting to change individual behaviour through taxation or health campaigns which were considered misguided and patronizing (#11, −1; #38, 0; #6, 0; #14, 0). Respondents instead emphasized the need for personal responsibility to pursue a healthy lifestyle (#9, −2; #20, −2), and the recognition of the importance of cultural context in designing health interventions (#39, −3; #28, +1; #13, +1; #15, +2).

### 3.2 Factor 2‐ Protect and care for the community

Factor 2 respondents emphasized the need for adequate health and care services to look after the elderly and vulnerable within the community, specifically those struggling on low incomes, who were considered deserving of being looked after by the state (#15, +5; #6, +4; #13, +3; #38, +2). To achieve this, respondents favoured the expansion of health and care services and providing local people with the financial means to live a healthy life (#4, −5; #5, −4; #33, +1; #19, +1). Additionally, respondents had great concern for the plight of poorer local people (as opposed to those who have moved into the community), whom they felt should be provided with enough money to access amenities and services considered crucial to living a healthy life, but which were unattainably expensive within the community (#34, +5; #31, +3, #14, +2, #17, +2).

There is a lot of poverty on the islands, and people do struggle, and when there’s no work people don’t have money to pay for their basic needs. And unemployment is probably quite high, because the opportunity probably isn’t there for people, they’re either not qualified or there’s just not the jobs. And to live on benefits really doesn’t meet all their needs I don’t think’. (Respondent 57)

In contrast to the care shown for low‐income ‘locals’, those moving into the community were not afforded the same concern and were further considered detrimental to the health of the community. It was claimed the social services department of the council had chosen to rehouse so‐called ‘social care people’ (Respondent 9) in social housing within the community, against the wishes of residents. Respondents holding this view favoured measures to mitigate the negative influence of poorer incomers on the health of the local population. This took the form of restricting their ability to move to the community (#36, −1; #24, +2) and implementing punitive measures to crack down on the drugs and crime that they are claimed to have introduced to the community, and which were detrimentally affecting the health of local people (#20, +4; #3, +4; #22, +3).

They are a very, very bad influence in the local community here, all these drug addicts and things that were almost unknown here before, and the knock‐on effect that has… there are young people taking up drugs, and of course that’s bad for their health. And there are some people taking part in crime now. And it’s just bad all round. And then it’s got to the stage now that people are locking their cars, locking their doors, things that never happened before. It’s causing anxiety amongst people, as well as all the diseases and sexual promiscuity, and all the rest of it. (Respondent 9)

Finally, respondents on Factor 2 claimed that empowering the community to play more of a role in local governance was having a detrimental effect on the health of residents, due to the social division it was perceived to cause (#27, −5; #37, −3; #1, −2; #2, −1). This belief also manifested itself in a negative perception of community‐based development strategies—whether economic (#18, −2; #32, −1), cultural (#28, −2; #32, −1) or environmental (#25, −3; #30, −3)—with regard to their impacts upon health.

### 3.3 Factor 3 Redistribution of resources

Respondents defining Factor 3 favoured a societal approach to redistributing resources through taxation (#5, +5) and redirecting political priorities to provide affordable goods and public services (#35, +4; #31, +3; #26, +3; #7, +2). These are seen to mitigate the high cost of living a healthy life within rural communities (#19, +5; #34, +4; #17, +3; #31, +3), as well as the negative health effects of poverty.

They can’t heat their houses… That’s key. And if we’re saying to be well you need to be comfortable, warm and dry, then that is critical, for everybody. So for people who don’t have good enough wages, there has to be a system that will enable them to be in a warm, dry place, and then to be able to buy proper food. (Respondent 20)

Tax revenues were to be spent on health and care services (#6, +4; #13, +2; #14, +2; #15, +1) to support people, but not social care services (#38, −2) or public health campaigns which were perceived to patronize and punish poorer people instead of altering their circumstances (#22, +1; #12, 0; #21, 0; #9, −1). Alternative fiscal or punitive solutions, including financial disincentives to crime or negative health behaviours, were also claimed to further disadvantage poorer people by increasing their cost of living (#4, −5; #20, −4; #3, −1) and detrimentally affecting their health. While a lack of service provision was considered to affect the entire community, the difficulty of accessing geographically distant services was seen to disproportionately affect poorer people.

One of the hardest things for people is to access healthcare here… The bus service has been cut so if they don’t drive or they have to rely on taxis, but the people who don’t drive are often the ones who have the least money, so therefore it’s difficult for them to find the money to spend on a taxi to come here. So it’s very difficult for some to access, or just to attend the doctor’s surgery. (Respondent 49)

This focus on state intervention was mirrored by less emphasis being placed on individual control (#40, −4; #23, −3), or the value of community culture and identity (#1, −4; #32, −3, #28, −2). While respondents holding this view did not feel strongly about the effects of local decision‐making mechanisms (#27, −3; #37, −1; #2, 0), they emphasized the need for the accessible provision of local services through the redistribution of public money towards the local community (#6, +4; #13, +2). This reflects the allegation that decisions related to the provision of public services were not adequately taking account of the needs of the community, with money being spent elsewhere instead.

The Council in Stornoway [main town and capital of the Outer Hebrides, located in the ‘Northern Isles’] won’t spend the money [here] in the Southern Isles. There’s a massive distinction between the Southern Isles and the Northern Isles. The bulk of the population are there [in the Northern Isles], but per capita less is spent here. (Respondent 28)

The redistribution of resources contained both a fiscal and political emphasis, but ultimately resulted in the same outcome, the wider provision and accessibility of public services within the community.

### 3.4 Factor 4‐ Investing in people

Respondents defining Factor 4 saw the cause of poor health in the community deriving from individuals engaging in negative and often self‐destructive health behaviours due to low self‐value and self‐esteem. Consequently, they favour investments in people to improve their self‐value to the point where they take control of their own health improvement through better lifestyle choices, largely through community‐based solutions (#34, +5; #16, +4; #33, +2). Public health campaigns were sometimes considered misdirected and potentially harmful in rural communities (#26, +3; #12, 0) with concurrent campaigns on alcohol and tobacco leading to individuals instead drinking and smoking to excess in their households, not curbing either behaviour and increasing their social isolation (#22, +3; #21, +1; #3, −1).

The provision of employment was considered to directly address the social and economic precursors of poor self‐value through reducing isolation and earning an income (#16, +4; #33, +2), which was considered vital to allow individuals to afford to live healthy, sociable lives (#34, +5; #19, +5; #10, +3).

If you don’t have money you can’t afford to be healthy really. You can’t afford to do anything. And your mental health will decline rapidly if you don’t have a good job. It’s key to good living is having a good job. (Respondent 15)

The importance of having enough money to pursue a healthy life was recognized in respondents disagreeing with punitive punishments for the least well‐off (#4, −5; #20, −4). However, they opposed financial benefits which did not derive from earned income, as they lacked the added feeling of usefulness and physical labour which often accompanied employment (#40, −4; #23, −3).

Isolation was also targeted through attempts to repopulate the community and the promotion of physical interactions over the increasingly pervasive digital alternatives (#24, +2; #36, −5; #7, −4). Such interactions were promoted through engagement in sports and exercise facilities, whether indoor or outdoor (#10, +3). While this encompassed the provision of specific facilities, there was also a large emphasis on protecting and enhancing the local environment, predominantly through placing restrictions on polluters (#25, +1; #30, 0; #17, −2; #8, −3).

Providing spaces and opportunities for leisure, recreation and other community activities. We’ve got a few but I don’t think we have enough. I don’t think there’s enough encouragement for young people to go outside and go play as much as there was when I was younger. (Respondent 24)

While respondents believed that overcoming social and economic challenges would improve self‐value and health behaviours, it was acknowledged that not everyone could pursue such opportunities due to physical or mental health issues. For these individuals, there was a need for investment in health‐care services, and specifically social care and counselling services to assist those struggling with mental health problems (#13, +4; #38, +2, #15, +2; #6, +1). The need for these services to be anonymous was also emphasized (#11, +4).

## 4 DISCUSSION

This study aimed to understand the shared perspectives within one remote‐rural island community on how to improve rural health. This section will discuss the nature of factors, and their convergences and divergences, in relation to rural health literature. The particular circumstances of the study locale are then considered with regard to how they improve our understanding of local perspectives on rural health improvement.

### 4.1 The factors

Factor 1 favours a conception of a vibrant rural community, considering health in terms of both the local economy and population, through perceiving a virtuous cycle of employment, education and population retention. While good jobs and business development opportunities were perceived to deliver direct benefits to those involved, indirect benefits to the whole population were realized through the perceived sustainability of the local culture. In many ways, Factor 1 represents a vision of community independence, resisting external (especially public sector) ‘interference’ and seeking to build a sustainable micro‐economy for the benefit of local people. However, demands for public sector education provision and subsidies for fuel and transport may betray such perceived autonomy. This favouring of more ‘upstream’ social determinants of health mirrors the emphasis placed on the importance of ‘Broader social structures’ to rural health outcomes—‘The most frequently identified social determinants of health outcomes are income, education, housing, work status and type, in/exclusion, access to social resources and health services, and a range of environmental factors’.^2^

Factor 2 focuses on providing care and support for elderly and vulnerable residents, especially those on low incomes, considered ‘deserving’ of support from the state. This perspective may be characterized as a more traditional, ‘downstream’ approach to providing publicly funded rural health‐care while curbing individual negative health behaviours through financial disincentives and punitive measures.^24^, ^25^ Factor 2 was also broadly resistant to change in the community. Incomers to the island were viewed with suspicion and derision, while being ‘othered’ from more deserving ‘local’ people. Similarly, major structural changes to power dynamics within the community were opposed, with respondents preferring things to stay as they are, or as they were. Therefore, this factor broadly reflected the tendency within remote‐rural communities to be resistant to changes in health‐care provision due to uncertainty as to how it may affect the community's sustainability.^13^

Factor 3 adopted a broad societal approach to addressing inequalities which exacerbate the disadvantages of poorer and more vulnerable members of the community, and society at large. These inequalities were considered both financial and geographical in nature, requiring fiscal and political solutions to ensure that resources were being invested in addressing them. This political aspect relates to the consideration of ‘Broader health systems’,^2^ outlining how decisions taken at ‘higher’ levels of the health sector can affect rural service provision. The fiscal consideration mirrors a finding of a similar study into public perspectives on improving health in low‐income urban communities.^10^ In that study, Factor S‐3 (labelled ‘Redistribution’) favours ‘fundamental, structural changes [in the] distribution of income, wealth and power’^10^ through taxation to reduce societal inequalities and their negative effects on health. In this sense, both urban and rural respondents perceived fundamental changes in political decision making as necessary to improve health.

In contrast to this structural perspective, respondents defining Factor 4 saw the solution to improving rural health as lying within the community itself. Specifically, respondents sought to integrate and empower individuals within the community, through social interaction, employment, financial independence and community networks. The importance of locally defined care and support services was emphasized, intended to support and engage residents to the point where their self‐value (and financial capacity) allowed them to adopt positive health behaviours and confidently play an active role in the community. This theme aligns with the theoretical conception of ‘The rural locale’ in seeing health outcomes ‘result from the complex interplay of social processes and relations that shape what people do in these places and how they connect to others’.^2^ It also shares similarities with another factor emerging from low‐income communities (labelled ‘Paternalism’), favouring the provision of ‘supportive frameworks… to enable people in low‐income communities to make better choices’.^10^

### 4.2 Consensus and divergence between factors

The provision of amenities and services was a strong theme in each factor, emphasizing socio‐economic interventions such as employment, education and housing (F1), institutional health services (F2, F3) and community‐based care (F4). The emphasis placed on their provision reflects previous research indicating the role of geographical isolation in ‘inequalities of resource allocation’^1^ and the need to address a lack of amenities and services to improve rural health.^2^, ^4^

Addressing rural poverty was an overarching theme of the findings, with the four factors representing different strategies for improving material conditions and financial stability for the poorest residents.^1^ As well as reducing the psychological stress associated with poverty, money was considered important to allow local people to buy healthy food and access amenities, such as exercise and social events, which contributed to both mental and physical health outcomes. Structural solutions such as increased employment (F1, F4) and state provision (F2, F3) were favoured over improving access to financial products and services and financially rewarding improved health behaviours, which were considered short‐term and piecemeal. This consensus was also apparent in low‐income urban communities,^10^ with respondents emphasizing the perceived importance of financial security for health outcomes across the urban/rural divide.

Reflecting on the above sections, respondents’ perspectives broadly converge with theories pertaining to rural health improvement, with the four factors emphasizing different aspects of a multifaceted approach to addressing ‘poverty, discrimination, inequality, [and] inequalities of resource allocation’.^1^ While Factor 2 retained a traditional ‘downstream’ focus on health‐care provision, the other three factors emphasized the importance of more ‘upstream’ interventions for rural health. The recognition of the role of social determinants of health—including employment and education (F1), societal structures (F3) and social and community networks (F4)—in improving health outcomes underlines the importance of including such aspects in future public engagement and coproduction exercises.

The similarities of factors to a theoretical framework for analysing rural and remote health^2^ indicate an awareness among ‘lay’ respondents of the ways in which health can be improved. Thus, while communities can contain complex and multiple differences of opinion regarding health interventions,^20^ and engaging them in decision making can be ‘messy’,^6^ each of the outlined perspectives may contribute to informing a comprehensive approach to improving rural and remote health outcomes.

### 4.3 Local participatory mechanisms

This research was situated on the community‐owned South Uist Estate as part of a broader study of the role of community landownership in improving rural health.^26^ Local power structures are reconstituted following a community land‐buyout^34^ as the estate holds significant influence over social and economic conditions within the community, as well as involvement in service provision.^26^, ^28^ Such increased community empowerment can be used by ‘individuals, groups and collectives in their actions to create, maintain or challenge systems and current ways of doing things’,^2^ which can improve health. Indeed, respondents to the study on low‐income communities explicitly advocated for the devolution of ‘decision making responsibility’^10^ to improve health. Thus, this income‐generating, democratically governed local organization, accountable to the welfare of residents, may be well placed to elicit and act upon public perspectives for the improvement of local health outcomes.

However, despite respondents across all factors criticizing externally mandated policies for being misdirected and not fully understanding local circumstances or context‐specific needs, the role of community‐based power structures was viewed with ambivalence by respondents, and in the case of Factor 2, perceived to negatively affect health. While the period since the 2007 buy‐out of the South Uist Estate has seen a significant growth in employment, business development and capital expenditure on the estate,^35^, ^36^ it has also been marked by significant social division regarding the way in which the estate is managed and governed^37^; eroding faith in the ability of the community to effectively function as a level of authority.^26^ Thus, while opposing the centralization of services, and broadly believing in the ability of local people and institutions to improve health, the residents of the South Uist Estate did not currently consider the community landowner the best vehicle through which to do so.

### 4.4 Limitations

Q methodology does not enable claims to be made about the representativeness of the perspectives identified. However, this could be explored through sequencing survey methods.^38^ Alternatively, the same statement set could be used in other locations which would be an interesting avenue of future study.

## 5 CONCLUSION

This study identified four shared perspectives on how to improve rural health. For the first time, public perspectives are explored that go beyond the provision of health​‐care to also consider how rural health could be improved by acting on the social determinants of health. In general, respondents perceived ‘solutions’ relating to the latter as playing a significant role in improving rural health, emphasizing the importance of including such options in future studies and coproduction activities. Importantly, this work highlights that public perspectives on rural health improvement are not homogenous within or between communities and should not be treated as such.^20^ This poses a challenge to health providers in eliciting and understanding diverging perspectives and designing appropriate interventions in disparate rural and remote communities, with implications for rural health policy and practice.^13^

Nevertheless, divergent opinions should not be perceived as a barrier to effective public engagement in the development of effective health policy. In our study, the use of Q methodology enabled the identification of areas of agreement among the divergent perspectives. For instance, there was shared recognition that providing access to services and amenities in rural communities, and addressing rural poverty, is important for improving the health of residents. The ability to explore the views of local residents in relation to problems of poor health on the island, and identify areas of agreement, can act as a positive starting point for discussions around the design and development of tailored, place‐based and locally acceptable solutions. The relative success of such solutions in improving health could form the focus of future research on this topic.

Although top‐down health policies may appear misdirected or suffer from a lack of rural or island ‘proofing’, respondents also recognized the importance of continued public sector health‐care provision. Within the remote‐rural island community which formed the focus of this study, respondents sought independence and autonomy in designing solutions, though the specific local power dynamics involving the community‐owned estate meant they did not favour the involvement of this particular endogenous actor.

Further research is required to understand the complex relationship between power and health in rural communities to contribute to our understanding of how community empowerment can affect rural health, dependent upon its effective wielding. In addition, research should focus on how community‐based organizations can best collaborate with local or national government in the design of health interventions for the benefit of residents’ health. We recommend the wider use of Q methodology within rural health research for its ability to engage local people in conversations around health provision and identify divergences and convergences in shared perspectives.

## CONFLICT OF INTERESTS

The authors have no conflict of interest.

## Data Availability

The data that support the findings of this study are available from the corresponding author upon reasonable request.
